# Longitudinal Examination of Body Mass Index and Cognitive Function in Older Adults: The HELIAD Study

**DOI:** 10.3390/nu15071795

**Published:** 2023-04-06

**Authors:** Ismini Grapsa, Eirini Mamalaki, Eva Ntanasi, Mary H. Kosmidis, Efthimios Dardiotis, Georgios M. Hadjigeorgiou, Paraskevi Sakka, Nikolaos Scarmeas, Mary Yannakoulia

**Affiliations:** 1 Department of Nutrition and Dietetics, Harokopio University of Athens, 17671 Athens, Greece; ismenegrapsa@gmail.com (I.G.); emamal@hua.gr (E.M.); e.ntanasi@hotmail.com (E.N.); 2 1st Department of Neurology, Eginition Hospital, Medical School, National and Kapodistrian University of Athens, 11528 Athens, Greece; ns257@cumc.columbia.edu; 3 Laboratory of Cognitive Neuroscience, School of Psychology, Aristotle University of Thessaloniki, 54124 Thessaloniki, Greece; kosmidis@psy.auth.gr; 4 Department of Neurology, Faculty of Medicine, University of Thessaly, 41500 Larissa, Greece; edar@med.uth.gr; 5 Department of Neurology, Medical School, University of Cyprus, Nicosia 2408, Cyprus; hadjigeorgiou.georgios@ucy.ac.cy; 6 Athens Association of Alzheimer’s Disease and Related Disorders, 11636 Maroussi, Greece; vsakka@ath.forthnet.gr; 7 Taub Institute for Research on Alzheimer’s Disease and the Aging Brain, The Gertrude H. Sergievsky Center, Department of Neurology, Columbia University, New York, NY 10032, USA

**Keywords:** obesity, weight loss, body mass index, cognitive functioning, cognitive decline, older adults

## Abstract

Given the increase in the aging population and thus in the prevalence of dementia, the identification of protective factors against cognitive decline is necessary. In a cohort of 1076 non-demented adults ≥ 65 years old (59.7% women) from the HELIAD study, we assessed whether changes in body mass index (BMI) were associated with changes in cognition over a 3-year follow-up period separately for those ≤ 75 and >75 years old. We identified six BMI trajectory groups based on participants’ BMI status at baseline and at the first follow-up visit; normal to normal BMI was the reference group. Major cognitive domains were evaluated, and a composite index reflecting global cognition was calculated. In participants aged ≤75 years, weight loss—moving from obesity to overweight or normal BMI—was associated with less decline in the memory composite score over time (β = 0.141; *p* = 0.035), while 3-year maintenance of a BMI ≥ 25 kg/m^2^ was related to greater reduction in the visuospatial composite score over time (β = −0.093; *p* = 0.020). Regarding participants aged >75 years, 3-year maintenance of a BMI ≥ 30 kg/m^2^ contributed to a slower rate of decline in the memory composite score over time (β = 0.102; *p* = 0.042), whereas weight loss—from overweight to normal BMI—was associated with a decreased attention/processing speed composite score longitudinally (β = −0.275; *p* = 0.043). Our findings indicated that the association between changes in BMI and cognitive functioning was modified by age. Weight management may have the potential to delay cognitive decline in older adults.

## 1. Introduction

With the aging of the population, the frequency of cognitive decline is increasing, and neurodegenerative diseases such as Alzheimer’s disease (AD) and other types of dementia are becoming increasingly common [1,2]. There are over 55 million people worldwide living with dementia, and this number will rise to 131.5 million by 2050 [2,3]. To date, there are no effective treatments widely available [4,5]. Therefore, the prevention of cognitive impairment and dementia is becoming necessary, and research activity is focused on investigating modifiable risk factors including energy balance and obesity.

The negative association between obesity in middle age and cognitive functioning has been well investigated [6,7]. Midlife obesity appears to be a risk factor for dementia in longitudinal studies with extended follow-up [8,9,10]. However, in older adults, the picture is more complicated, and the association between body weight and cognitive health remains unclear. In a representative sample of 3035 community-dwelling older adults, higher body mass index (BMI) and waist circumference were associated with a slower rate of cognitive impairment over a 7-year follow-up period [11]. In addition, weight loss has been related to an increased risk of cognitive decline and a higher risk of dementia in longitudinal studies of community-dwelling adults over 65 years of age [12,13]. Conversely, in a population-based cross-sectional study of 1949 participants aged ≥65 years, overweight and obesity were associated with worse cognitive performance compared with BMI < 25 kg/m^2^ [14]. Furthermore, intentional weight loss through diet may improve cognitive functioning in older adults with obesity, according to evidence from randomized controlled trials [15,16].

The inconsistency of findings can be attributed to certain methodological limitations of the studies. Unfortunately, studies focused on older adults often ignore the heterogeneity that characterizes this age group. It is important to recognize that there are differences in age-related changes that take place in the youngest-old (e.g., <70 years) and the oldest-old individuals (e.g., ≥85 years) [17], which may also modify the association between obesity and cognitive decline. Another crucial point is that some previous studies have performed a crude assessment of cognitive functioning using a limited range of cognitive tests; in particular, the assessment of cognitive functioning was based solely on the Mini Mental State Examination [12,18]. In order to clarify the relationship between changes in body weight and changes in cognitive functioning in older adults, it is important to study multiple cognitive domains through a comprehensive neuropsychological assessment. Finally, another factor that contributes to the inconsistency of findings across studies concerns the lack of adjustment for important confounders such as apolipoprotein E (APOE) ε4 allele as a marker of genetic predisposition and years of education as a marker of cognitive reserve [19].

Thus, the aim of the present analysis is to fill in the gap in the existing literature and address most of the limitations of the previous studies, examining whether changes in BMI are related to changes in cognitive performance over time in a representative cohort of older adults. We evaluated this potential association separately in participants aged ≤75 years and >75 years. This methodological approach is particularly important for understanding how age may potentially modify the association between changes in body weight and cognitive decline. In addition, we comprehensively assessed the cognitive function of participants using a detailed battery of neuropsychological tests, and we adjusted for several potential confounders that have been previously neglected.

## 2. Materials and Methods

### 2.1. Study Design and Participants

The Hellenic Longitudinal Investigation of Aging and Diet (HELIAD) study is a population-based, multidisciplinary study of Greek older adults examining the epidemiology of AD, other types of dementia, mild cognitive impairment (MCI), and other aging-related neuropsychiatric conditions. The baseline evaluation of the HELIAD study started in 2011. As this is a population-based study, participants were randomly selected from community-dwelling individuals over 65 years of age. In particular, participants were selected through municipality rosters from a suburb of Athens, Maroussi, and a city in central Greece, namely Larissa, including the surrounding areas, both urban and rural. Written informed consent was obtained from all individuals prior to their participation in the study. The study was approved by the Institutional Ethics Review Boards of the University of Thessaly and the National and Kapodistrian University of Athens. Detailed information was collected in face-to-face interviews by adequately trained health professionals (neurologists, neuropsychologists, and dieticians), collecting a series of lifestyle and other information. Participants’ caregivers were involved in providing information whenever needed. Extensive details of the study design and data collection have been published previously [20,21,22,23,24,25]. Based on participants’ preferences, sessions were carried out at day-care centers for older adults, the participants’ homes, or municipal public health clinics. The evaluations lasted 2.0–2.5 h per participant. Participants are reevaluated approximately every 3 years. Baseline assessments and consensus diagnosis are repeated at each follow-up. Overall, two full evaluations have been completed to date (the baseline and the first follow-up). These evaluations were included in the present work.

### 2.2. Anthropometry

Participants were dressed in light clothing, and their body weight was measured to the nearest 0.5 kg. Height was measured to the nearest 0.5 cm in bare feet and with the participant’s head in horizontal Frankfort plane. Body weight and height were measured using a mechanical column scale with height rod (SECA 700). BMI was calculated as weight in kilograms divided by height in meters squared (kg/m^2^). Body weight status of the participants was assessed based on the World Health Organization’s BMI cut-off points: <18.5 kg/m^2^ as underweight; 18.5–24.9 kg/m^2^ as normal weight; 25.0–29.9 kg/m^2^ as overweight; and ≥30.0 kg/m^2^ as obesity [26]. Anthropometric indices (body weight and height) were measured at baseline as well as at the first follow-up visit.

### 2.3. Neuropsychological Evaluation

At both visits, a comprehensive neuropsychological evaluation was conducted, assessing all major cognitive domains with a battery of neuropsychological tests. The duration of the neuropsychological evaluation was about 1 h per participant. Specifically, trained neuropsychologists assessed attention/processing speed, executive functioning, memory, language, and visuospatial ability using the neuropsychological tests listed as follows: Attention and Information Processing Speed (Trail Making Test-Part A [27]), Executive Functioning (Trail Making Test-Part B; Graphical Sequence Test; Anomalous Sentence Repetition; Motor Programming [28]; months forwards and backwards), Non-verbal and Verbal Memory (Greek Verbal Learning Test [29]; Medical College of Georgia Complex Figure Test [28]), Language (subtests of the Greek version of the Boston Diagnostic Aphasia Examination short form, namely the Boston Naming Test-short form, and selected items from the Complex Ideational Material Subtest to assess verbal comprehension and repetition of words and phrases [30]; a semantic and phonological verbal fluency test [31]), and Visuoperceptual Ability (Medical College of Georgia Complex Figure Test copy condition [28]; Judgment of Line Orientation [32,33] abbreviated form; Clock Drawing Test [28,34]).

Based on the mean and standard deviation values of participants without MCI or dementia, participants’ raw scores on each neuropsychological test were converted into z-scores. Then, z-scores of individual neuropsychological tests were averaged to derive composite scores for the following cognitive domains: executive functioning, memory, language, attention/processing speed, and visuospatial ability. Subsequently, these domain composite z-scores were averaged to compute a global cognition score. Higher z-scores indicated better cognitive performance. Speed scores were reversed so that higher task-completion times yielded lower scores, indicating poor performance.

### 2.4. Neurological Evaluation and Clinical Diagnosis

The neurological evaluation was performed by certified neurologists at both visits. All information obtained was reviewed, and diagnoses were determined after consensus meetings of neuropsychologists and neurologists. Dementia was diagnosed according to the Diagnostic and Statistical Manual of Mental Disorders, Fourth Edition, Text Revision (DSM-IV) [35]. The diagnosis of MCI was based on the Petersen criteria [36,37]. At both visits, the questions used to diagnose dementia and MCI were exactly the same.

### 2.5. Assessment of Depressive Symptoms/Depression

The Geriatric Depression Scale, a 15-item self-report questionnaire regarding depressive symptoms in the past week, was used to assess depressive symptoms at baseline [38,39]. Furthermore, when neurologists examined participants, they recorded current medications and assessed whether participants met DSM-IV criteria for depression [35]. Participants who scored ≥6 on the Geriatric Depression Scale [39] and/or were receiving antidepressant medication and/or had been diagnosed with depression were considered to suffer from depression.

### 2.6. Other Variables

Demographic variables of interest such as age and education level (both in years) as well as sex were recorded. Furthermore, neurologists recorded any chronic diseases reported by participants at the beginning of the study; this information was included in a variable indicating the sum of comorbidities. Finally, a peripheral blood sample was collected for APOE genotyping. APOE genotyping was performed in genomic DNA extracted from blood buffy coat, using QIAamp DNA Blood Mini Kits (Qiagen, Venlo, The Netherlands). The method used for the genotyping was polymerase chain reaction-DNA sequencing, carried out with LightCycler 2 (Roche Diagnostics AG, Basel, Switzerland) and using the LightMix TIB MOLBIOL reactors.

### 2.7. Statistical Analyses

Continuous variables are expressed as mean ± standard deviation and categorical variables as relative frequencies (%). Pearson’s χ^2^ tests and *t*-tests were used to evaluate differences between groups. Participants with dementia at baseline (*N* = 83) and participants who were underweight (*N* = 7) were excluded from the analyses.

Generalized estimating equations (GEE) models were performed to examine whether changes in BMI were associated with rates of change of cognitive composite scores over time. Analyses were conducted separately for participants aged ≤75 years and >75 years. Individuals were categorized into one of six BMI trajectory groups based on their BMI at baseline and at the first follow-up visit: normal to normal BMI (reference), normal to overweight or obesity BMI, overweight to normal BMI, overweight to overweight or obesity BMI, obesity to overweight or normal BMI, and obesity to obesity BMI. First, the global cognition score was the dependent variable, and time to follow-up (in years from baseline assessment), BMI trajectory group, and BMI trajectory group × time interaction term were the predictors. We then repeated GEE analyses using the individual cognitive domain scores (executive functioning, memory, language, attention/processing speed, and visuospatial ability) as dependent variables. The results represent the estimates (β coefficients for linear GEE models) of the BMI trajectory group × time interaction term. In our main models, potential confounders included years of education, sex, and APOE-ε4 carriage. Sex and APOE-ε4 carriage (no ε4 allele vs. one or two ε4 alleles) were used as dichotomous variables; education was treated as a continuous variable. Finally, we conducted supplementary analyses; we repeated the above analyses using depression and the sum of comorbidities as additional confounders. The sum of comorbidities was treated as a continuous variable, while depression was treated as a dichotomous variable.

Statistical analyses were conducted using the SPSS software version 26.0 (SPSS, Chicago, IL, USA). Statistical significance was set at *p* ≤ 0.05.

## 3. Results

Among 1984 participants at baseline, 880 were lost to follow-up. Volunteers who completed both evaluations were younger (73.2 ± 5.0 mean age ± standard deviation for those who returned for the second visit vs. 74.7 ± 5.9 for those who did not return for the second visit; *p* < 0.001). Education level did not differ between participants with complete data and those who did not return for the second visit (*p* = 0.127). Moreover, individuals with complete data had a higher global cognition score compared with those who did not complete both evaluations (−0.08 ± 0.75 vs. −0.29 ± 0.79, respectively, *p* < 0.001).

In total, 1076 individuals without dementia at baseline who completed the initial assessment and attended the first follow-up visit were included in the analyses. The mean duration of the follow-up period was 3.0 ± 0.8 years. When the sample was divided based on age, we observed significant differences between the two age groups. Table 1 summarizes demographic and clinical characteristics and cognitive composite scores at baseline as well as anthropometric characteristics at baseline and at the first follow-up visit for participants aged ≤75 years and >75 years. Individuals aged ≤75 years were more educated (*p* < 0.001) and had higher cognitive composite scores (*p* < 0.001 for all comparisons) compared with individuals aged >75 years. Furthermore, participants aged ≤75 years were more likely to be women (*p* < 0.001). Finally, participants aged ≤75 years were more likely to have one or two ε4 alleles (*p* = 0.001). Other characteristics such as BMI, body weight, depression, and sum of comorbidities did not differ between the two groups (Table 1).

Table 2 shows the association between BMI trajectories and rates of change of cognitive composite scores over time by age group. In both age groups, changes in BMI did not appear to affect rate of change of the global cognition score over time. However, in relation to memory, in participants aged ≤75 years, decrease in BMI—from obesity to overweight or normal BMI—was associated with less decline in the memory composite score over time (14.1% of a standard deviation less decline for each additional year of follow-up; *p* = 0.035) compared with participants aged ≤75 years with a stable normal BMI. Conversely, participants aged >75 years who remained in the obesity range at both assessments had a slower rate of decline in the memory composite score over time (10.2% of a standard deviation less decline for each additional year of follow-up; *p* = 0.042) compared with participants aged >75 years with a stable normal BMI.

As far as attention/processing speed is concerned, in individuals aged ≤75 years, BMI trajectories did not seem to affect rate of change of the attention/processing speed composite score over time. On the contrary, individuals aged >75 years who lost weight, thus moving from the overweight to the normal BMI range, had a greater decline in the attention/processing speed composite score over time (27.5% of a standard deviation reduction for each additional year of follow-up; *p =* 0.043) than those who remained at a normal BMI. Furthermore, participants aged ≤75 years in the overweight range during the 3-year follow-up period or in the overweight range at baseline and obesity at the follow-up visit showed greater reduction in the visuospatial composite score over time (9.3% of a standard deviation reduction for each additional year of follow-up; *p =* 0.020) in comparison with participants aged ≤75 years with a stable normal BMI. No such association was detected in participants aged >75 years (Table 2).

We further examined the association between BMI trajectories and rates of change of cognitive composite scores over time by adding depression and the sum of comorbidities as confounders in the models. The main results did not change apart from the association between weight loss—from overweight to normal BMI—and reduction in the attention/processing speed composite score over time in participants aged >75 years, which became marginally significant (β = −0.269; *p =* 0.061). We also found that, in participants aged ≤75 years, decrease in BMI—from overweight to normal BMI—was associated with a slower rate of decline in the memory composite score over time (10.3% of a standard deviation less decline for each additional year of follow-up; *p* = 0.036) (results not shown).

## 4. Discussion

The present longitudinal study investigated the potential association between changes in BMI and cognitive functioning over time in Greek community-dwelling older adults. Our findings indicated that in individuals aged ≤75 years, weight loss—moving from obesity to overweight or normal BMI—is associated with less decline in memory performance over time, while 3-year maintenance of a BMI ≥ 25 kg/m^2^ is related to decreased visuospatial ability longitudinally. On the contrary, obesity was associated with less memory decline over time in participants aged >75 years, whereas weight loss—from overweight to normal BMI—contributed to a faster rate of decline in the attention/processing speed domain over time.

Overall, the present study indicated that obesity and changes in BMI were associated with changes in cognitive performance over time, and these associations were modified by age. Our results are in the same direction as a previous longitudinal cohort study that examined the relationship between incident dementia and obesity and also found that this relationship was age-dependent. Specifically, there was U-shaped association between dementia and BMI in participants aged <76 years, while participants aged ≥76 years with increased BMI had a lower dementia risk [40]. The differences found in the oldest-old and youngest-old individuals may be due to the different characteristics that the two groups have; as age increases, older individuals have more chronic diseases and comorbidities, which may make them more susceptible to the negative effects of different conditions, including weight loss and/or decreased BMI. Thus, weight loss in youngest-old individuals with obesity may offer protection against obesity-related consequences, while weight loss in oldest-old individuals may contribute to further deterioration in their health status, making obesity appear protective.

In the younger age group, i.e., subjects aged ≤75 years old, we found that weight loss in those with obesity has a beneficial effect on the memory domain, while higher BMI (BMI ≥ 25 kg/m^2^) is related to a faster rate of impairment in visuospatial ability over time. Obesity has been associated with cognitive dysfunction in older people both cross-sectionally and longitudinally [41,42], while weight loss through dietary intervention has been found to enhance cognitive functioning in older adults with obesity [43]. Obesity is related to functional and structural abnormalities in the brain [19,44], which may lead to cognitive dysfunction. In specific, increased BMI has been linked to lower gray matter in the temporal lobe, occipital lobe, frontal lobe, and midbrain and decreased white matter integrity throughout the brain [19,45,46]. Furthermore, obesity leads to chronic low-grade systemic inflammation, which potentially causes neuroinflammation [47]. In particular, obesity increases levels of circulating free fatty acids, immune cells, and pro-inflammatory cytokines, which enhance blood–brain barrier permeability and ultimately enter the hypothalamus [47,48,49]. This leads to activation of the pro-inflammatory transcription factor NF-κB and the increased expression of pro-inflammatory mediators and cytokines, resulting in the development of neuroinflammation. Overall, neuroinflammation leads to synaptic remodeling, decreased neurogenesis, and neuronal apoptosis [47]. As a final point, obesity is a predisposing factor for a number of pathological conditions that themselves increase the risk of cognitive decline, such as type 2 diabetes mellitus and hypertension [19]. However, more studies are needed to elucidate the mechanisms among the youngest-old individuals.

Our analyses in participants aged >75 years demonstrated that 3-year maintenance of a BMI ≥ 30 kg/m^2^ is associated with less decline in memory performance over time, while weight loss—from overweight to normal BMI—is associated with reduction in the attention/processing speed composite score over time. These results are broadly consistent with previous longitudinal studies. For example, a 9-year longitudinal study found that participants ≥ 75 years with a BMI ≥ 25 kg/m^2^ had a lower risk of developing dementia than those with a normal BMI. Furthermore, a significant decrease (>10%) in BMI contributed to a 50% higher risk of dementia [50]. Moreover, another longitudinal study indicated that lower BMI in older adults was associated with a faster rate of cognitive decline [51].

This inverse association between cognitive impairment and obesity in older adults aged >75 years represents an obesity paradox, which could be explained by several pathways. First, the reverse causation hypothesis is a possible explanation. Dementia has a long preclinical phase. Weight loss due to progressive cognitive impairment and underlying pathological changes may begin years before the diagnosis of dementia. Weight loss can be the result of difficulty eating, loss of initiative, predementia apathy, impaired olfaction, and inadequate nutrition due to cognitive decline [44,52,53,54]. Second, weight loss may occur in the context of frailty [55]. It has been suggested that frailty contributes to the onset of dementia through inflammation and oxidative stress, although the exact mechanism remains unknown [56]. Therefore, weight loss in participants aged >75 years may be due to the preclinical stage of the disease or the coexistence of frailty, which is a possible explanation for the inverse association between obesity and cognitive decline that we found. Finally, during weight loss, adipose tissue is lost, and as a result, leptin levels may decrease. Leptin is a hormone secreted by adipose tissue, which is suggested to have a protective effect against cognitive decline, contributing to neuronal survival [52].

The present study has certain limitations. We used BMI as the index for assessing obesity, although it is well known that BMI does not reflect changes in lean and fat mass [57,58]. Another limitation of the present study concerns the fact that we did not assess whether the weight loss was unintentional (i.e., because of pre-existing disease) or intentional. Hence, including individuals with unintentional weight loss in the analyses may have attenuated the observed associations. Although we adjusted our models for potential confounders, the existence of other confounding variables not evaluated in this work (i.e., residual confounding) cannot be ruled out completely. Finally, the length of the follow-up period could be considered relatively short for excluding a reverse causality hypothesis given the long preclinical phase of dementia and the slow progression of the disease. However, the follow-up period in the HELIAD study is ongoing, and the completion of the third full evaluation (second follow-up visit) is expected.

The strengths of our study include the longitudinal design that allows us to investigate the cause-and-effect relationship between changes in BMI and cognitive impairment. Furthermore, our sample is representative of the population under investigation, enhancing the generalization of our findings. At the same time, only older adults were included in our study in contrast to other studies that used different age groups (e.g., individuals > 40 years) [59,60], and consequently, no firm conclusions could be drawn about this specific age group. Another important strength of our study is that analyses were conducted by age group, which allowed us to draw conclusions beyond the heterogeneity that distinguishes older adults. The lack of subgroup analysis of such a heterogeneous group may explain some of the inconsistencies in the results as reported in the existing literature. Another strength of our research is that a comprehensive neurological and neuropsychological evaluation was conducted by specialized personnel, while clinical diagnoses were determined by a multidisciplinary consensus expert team based on international criteria. Therefore, we were able to study all major cognitive domains. Anthropometric indices (body weight and height) were remeasured at the first follow-up visit and were not derived from self-reported information, which can often be unreliable. Finally, a large amount of data was collected through validated measures in the HELIAD study, allowing us to adjust our models for important confounders.

## 5. Conclusions

The findings of the present study suggest that weight loss in individuals with obesity aged ≤75 years contributes to a decelerated rate of decline in memory performance over time, whereas 3-year maintenance of a BMI ≥ 25 kg/m^2^ was associated with decreased visuospatial ability longitudinally. In contrast, in individuals aged >75 years, maintaining a BMI ≥ 30 kg/m^2^ was found to have a protective role against memory decline, while weight loss was associated with a faster rate of impairment in the attention/processing speed domain over the course of time. Thus, we suggest that BMI change should be considered in future studies and should be assessed by health professionals in clinical practice. At the same time, future research using diagnostic imaging techniques and predictive AD biomarkers and combining different methods of measuring adiposity should focus on the mechanisms by which obesity could affect cognitive functioning, while it is very important to consider whether weight loss occurs unintentionally or intentionally. In conclusion, our results may be of clinical relevance, as early, individualized interventions targeting modifiable risk factors such as obesity could reduce the risk of cognitive decline in older adults.

## Figures and Tables

**Table 1:** Demographic, anthropometric, and clinical characteristics and cognitive composite scores in participants aged ≤75 years and >75 years.

	≤75 Years Old (*Ν* = 715)	>75 Years Old (*Ν* = 361)	*p*-Value *
Sex (% females)	65.5%	48.2%	**<0.001**
Education (years)	8.7 ± 4.7	7.0 ± 5.1	**<0.001**
Body mass index (kg/m^2^)			
at baseline	29.0 ± 4.6	28.7 ± 4.2	0.365
at follow-up	29.2 ± 4.7	28.7 ± 4.4	0.104
Body weight (kg)			
at baseline	75.8 ± 12.9	75.0 ± 12.2	0.284
at follow-up	76.1 ± 13.2	74.8 ± 12.8	0.122
APOE-ε4 carriage (% carrying one or two ε4 alleles)	19.3%	12.6%	**0.001**
Depression (% yes)	20.4%	18.6%	0.517
Sum of comorbidities	2.0 ± 1.4	2.2 ± 1.5	0.094
Global cognition	0.20 ± 0.60	−0.44 ± 0.83	**<0.001**
Executive functioning	0.19 ± 0.63	−0.39 ± 0.86	**<0.001**
Memory	0.23 ± 0.83	−0.44 ± 0.90	**<0.001**
Attention/Processing speed	0.17 ± 0.87	−0.64 ± 1.30	**<0.001**
Language	0.25 ± 0.69	−0.34 ± 1.01	**<0.001**
Visuospatial ability	0.19 ± 0.62	−0.38 ± 1.12	**<0.001**

Continuous variables are presented as mean ± standard deviation. Categorical variables are presented as relative frequencies (%). * *p*-values were derived from *t*-tests or Pearson’s χ^2^ tests, exploring differences between participants aged ≤75 years and >75 years. Values in bold indicate statistically significant findings (*p* < 0.05).

**Table 2:** Results from generalized estimating equations that evaluated the association between BMI trajectories (independent variables) and differential rates of change of cognitive composite scores over time (dependent variables) in participants without dementia at baseline.

		≤75 Years Old		>75 Years Old	
	BMI Trajectories	β (±SE)	*p*-Value	β (±SE)	*p*-Value
Global cognition					
	Normal → Normal (reference)				
	Normal → Overweight or Obesity	0.012 ± 0.0285	0.680	0.004 ± 0.0578	0.949
	Overweight → Normal	0.044 ± 0.0352	0.210	−0.002 ± 0.0400	0.952
	Overweight → Overweight or Obesity	−0.029 ± 0.0216	0.178	−0.012 ± 0.0401	0.756
	Obesity → Overweight or Normal	0.027 ± 0.0519	0.600	0.018 ± 0.0420	0.665
	Obesity → Obesity	−0.024 ± 0.0227	0.282	−0.029 ± 0.0378	0.442
Executive functioning					
	Normal → Normal (reference)				
	Normal → Overweight or Obesity	0.006 ± 0.0396	0.880	0.059 ± 0.0620	0.338
	Overweight → Normal	0.078 ± 0.0516	0.129	−0.022 ± 0.0746	0.765
	Overweight → Overweight or Obesity	−0.011 ± 0.0249	0.657	0.017 ± 0.0472	0.725
	Obesity → Overweight or Normal	−0.001 ± 0.0476	0.990	0.059 ± 0.0495	0.231
	Obesity → Obesity	−0.001 ± 0.0269	0.980	−0.025 ± 0.0503	0.626
Memory					
	Normal → Normal (reference)				
	Normal → Overweight or Obesity	0.050 ± 0.0590	0.402	0.059 ± 0.0894	0.507
	Overweight → Normal	0.073 ± 0.0548	0.184	0.145 ± 0.0805	0.071
	Overweight → Overweight or Obesity	−0.001 ± 0.0396	0.984	0.046 ± 0.0485	0.340
	Obesity → Overweight or Normal	0.141 ± 0.0671	**0.035**	0.077 ± 0.0737	0.297
	Obesity → Obesity	0.017 ± 0.0396	0.665	0.102 ± 0.0501	**0.042**
Attention/Processing speed					
	Normal → Normal (reference)				
	Normal → Overweight or Obesity	−0.068 ± 0.0485	0.164	0.177 ± 0.1436	0.218
	Overweight → Normal	−0.005 ± 0.0518	0.918	−0.275 ± 0.1360	**0.043**
	Overweight → Overweight or Obesity	−0.053 ± 0.0347	0.128	−0.039 ± 0.0857	0.652
	Obesity → Overweight or Normal	−0.089 ± 0.1136	0.433	−0.118 ± 0.1153	0.306
	Obesity → Obesity	−0.066 ± 0.0363	0.068	−0.167 ± 0.0945	0.077
Language					
	Normal → Normal (reference)				
	Normal → Overweight or Obesity	0.021 ± 0.0428	0.625	−0.028 ± 0.0673	0.675
	Overweight → Normal	0.090 ± 0.0490	0.065	0.018 ± 0.0743	0.805
	Overweight → Overweight or Obesity	0.011 ± 0.0282	0.689	−0.001 ± 0.0587	0.987
	Obesity → Overweight or Normal	0.017 ± 0.0598	0.776	0.052 ± 0.0683	0.448
	Obesity → Obesity	−0.004 ± 0.0307	0.909	−0.015 ± 0.0607	0.808
Visuospatial ability					
	Normal → Normal (reference)				
	Normal → Overweight or Obesity	0.023 ± 0.0432	0.602	−0.153 ± 0.0792	0.053
	Overweight → Normal	−0.051 ± 0.0496	0.303	0.041 ± 0.0759	0.591
	Overweight → Overweight or Obesity	−0.093 ± 0.0398	**0.020**	−0.048 ± 0.0644	0.460
	Obesity → Overweight or Normal	0.025 ± 0.0736	0.734	−0.017 ± 0.0848	0.838
	Obesity → Obesity	−0.061 ± 0.0383	0.112	−0.040 ± 0.0595	0.503

SE, standard error; BMI, body mass index. All models were adjusted for years of education, sex, and apolipoprotein E (APOE) ε4 carriage. Values in bold indicate statistically significant findings (*p* < 0.05).

## Data Availability

The data are available from the corresponding author upon reasonable request.
